# Circulating inflammatory biomarkers as adjusted prognostic predictors of post-stroke cognitive impairment: a comparative meta-analysis of cytokines and lymphocyte-based ratio indices in prospective cohorts

**DOI:** 10.3389/fnagi.2026.1937870

**Published:** 2026-10-21

**Authors:** Burcu Yuksel, Begüm Çapa Tayyare, Nurullah Eryilmaz

**Affiliations:** 1 Kocaeli Vocational School of Health Services, Kocaeli University, Kocaeli, Türkiye; 2 Leuphana University, Luneburg, Germany

**Keywords:** inflammation, interleukin-6, ischaemic stroke, meta-analysis, neutrophil-to-lymphocyte ratio, post-stroke cognitive impairment, prognosis, systemic immune-inflammation index

## Abstract

**Introduction:**

Systemic inflammation is implicated in post-stroke cognitive impairment (PSCI). Two families of blood markers have been studied as predictors: cytokines (interleukin-6, C-reactive protein) and lymphocyte-based ratio indices from the full blood count, including the neutrophil-to-lymphocyte ratio, the systemic immune-inflammation index (SII) and the globulin-to-lymphocyte ratio (GLR). Existing syntheses pool unadjusted marker differences within a single family, so their relative adjusted associations with PSCI are unknown.

**Methods:**

We systematically reviewed strictly prospective cohorts reporting a covariate-adjusted association between a circulating inflammatory marker and PSCI. Estimates were harmonised to the odds ratio per one standard deviation (SD) increase, deriving SDs where needed from medians and interquartile ranges and rescaling category contrasts by category-spacing factors. The pre-defined families were compared by moderator test, with random-effects models and Hartung-Knapp inference. Reporting followed PRISMA 2020; risk of bias was assessed with QUIPS.

**Results:**

Sixteen studies were eligible; ten independent cohorts provided a harmonisable per-SD estimate. A one-SD increase was associated with higher odds of PSCI (odds ratio 1.60, 95% CI 1.25 to 2.05; I-squared 77%; prediction interval 0.79 to 3.23, including the null). The families did not differ: ratio indices, including the HALP (haemoglobin, albumin, lymphocyte, platelet) score, 1.61 (95% CI 1.15 to 2.26) versus cytokines 1.57 (95% CI 0.70 to 3.53); ratio of odds ratios 0.97 (95% CI 0.54 to 1.77), *p* = 0.92. Point estimates for the SII and GLR were higher than for other markers, but each rested on one or two cohorts, and the two-study SII prediction interval was wide. The pooled estimate was stable across leave-one-out and other sensitivity analyses, including an alternative conversion route and exclusion of the two cohorts assessing cognition within 2 weeks of stroke (1.61, 95% CI 1.19 to 2.18).

**Conclusion:**

Some prospective cohorts suggest that higher post-stroke inflammation-related blood biomarkers are associated with higher subsequent odds of cognitive impairment, but clinical and statistical heterogeneity is substantial. On this evidence (ten cohorts, almost all from single centres in East Asia), neither family can be shown to be more strongly associated with PSCI, no single marker can be prioritised, and clinical utility is not demonstrated.

## 1 Introduction

Stroke is a leading cause of acquired disability, cognitive impairment being one of its most serious consequences. Post-stroke cognitive impairment (PSCI) refers to any level of cognitive deficit that occurs after a clearly diagnosed stroke, including cases of mild, single-domain impairment as well as dementia. It affects a large number of survivors, the prevalence of which depends on the type of stroke, the assessment tool used and when the testing takes place (El Husseini et al., 2023; Sexton et al., 2019). PSCI hinders functional recovery and is linked to increased mortality as well as to a higher rate of stroke recurrence (Sandvig et al., 2023). Since there is a period between the onset of stroke and the development of established PSCI during which intervention is possible, it would be useful to have simple and inexpensive tools that can identify high-risk patients early after admission (Huang et al., 2025).

Systemic inflammation could serve as a connection between cerebral ischaemia and subsequent cognitive decline. When acute ischaemia occurs, it causes the release of damage-associated molecular patterns which in turn activate microglia and bring in innate immune cells from the periphery; neutrophils enter and cause the release of matrix metalloproteinase-9, leading to disruption of the blood-brain barrier, while lymphocytes play a role in tissue repair (Zhao et al., 2024; Bao et al., 2023). In this regard, two groups of circulating markers have been looked at. The first includes cytokines and acute-phase proteins, specifically interleukin-6 (IL-6), C-reactive protein and tumour necrosis factor-alpha. The second consists of indices obtained from a standard full blood count, for example the NLR, the platelet-to-lymphocyte ratio, the SII and the GLR. Since ratio indices are cheap and widely available, their use as predictors of PSCI has seen rapid development (Mao et al., 2025).

The body of evidence has three characteristics which restrict the way it can be interpreted. Firstly, most of the primary studies as well as the summaries based on them give the unadjusted differences in marker levels between people with and without PSCI, rather than providing estimates that take into account confounding factors such as age, education and the severity of the stroke. The most recent meta-analysis of lymphocyte-associated indices combined the weighted mean differences and found that levels were higher in those with PSCI, without giving a prognostic estimate that had been adjusted for confounders (Mao et al., 2025), and similar trends are observed in other comprehensive reviews of biomarkers (Kim et al., 2022; Jiang et al., 2025). Secondly, a number of the analyses regard PSCI as a common condition that is assessed at the same time as the marker, rather than treating it as an outcome that occurs after the exposure has been measured. Thirdly, the syntheses have so far been limited to one family of markers and therefore the two families have not been directly compared. Another aspect is that the large and well-adjusted body of research on IL-6 and CRP following a stroke has focused on vascular recurrence and major adverse cardiovascular events rather than on cognitive outcomes (McCabe et al., 2023). Since the cytokine evidence in the context of stroke appears to be mature, this does not apply to cognitive outcomes either, and the number of studies retrieved by using a marker's name is greater than the actual amount of evidence that is specific to cognition and which has been adjusted for confounders.

These gaps leave a basic question open: among circulating inflammatory markers measured after stroke, how strong are their adjusted associations with PSCI, and is there evidence that these associations differ between lymphocyte-based ratio indices and classical cytokines? Answering it requires estimates that are adjusted, that concern incident or longitudinally assessed cognition, and that are placed on a common scale so that markers can be compared rather than summarised in isolation.

We therefore conducted a systematic review and comparative meta-analysis designed to make that comparison possible. Inclusion was restricted to prospective cohorts reporting a covariate-adjusted association between an inflammatory marker and PSCI; every estimate was harmonised to the odds ratio per one standard deviation increase; the two pre-specified marker families, cytokines and lymphocyte-based ratio indices, were synthesised and tested formally against each other; and, where data permitted, the diagnostic effectiveness of the leading markers was examined as a secondary aim. The aim was comparative rather than confirmatory: the association of a single marker with PSCI is already documented, so the question here is how the candidate markers stand relative to one another on a common adjusted metric, and how robust the prospective evidence is.

## 2 Materials and methods

The review was carried out and presented in line with the PRISMA 2020 statement (Page et al., 2021); since the question is prognostic, the extraction was guided by the CHARMS checklist (Moons et al., 2014) and the risk of bias was evaluated using the QUIPS tool (Hayden et al., 2013).

### 2.1 Protocol

A protocol drafted a priori, outlining the criteria for eligibility, the two marker families, the restriction associated with the strictly prospective design and the analysis plan, was prepared before data extraction and can be obtained from the authors. The review was not entered in a public registry, a point which is recognised as a limitation.

### 2.2 Eligibility criteria

Eligibility was established using the PICOTS framework. The population included adults (that is, people aged 18 years or more with no maximum age limit) who had either an acute ischaemic stroke or a transient ischaemic attack; in the case of studies that only looked at intracerebral haemorrhage, eligibility was granted only if it was possible to separate out the figures for ischaemic events. Ischaemic events were the focus of the study since ischaemic and haemorrhagic strokes differ in terms of their pathophysiology and inflammatory response, and because the vast majority of the evidence regarding inflammatory biomarkers and PSCI relates to ischaemic stroke; combining the two types of stroke would have introduced clinical heterogeneity. The prognostic factor was a circulating inflammatory marker belonging to one of two pre-defined families. Family A (consisting of cytokines and acute-phase proteins) included IL-6, C-reactive protein and high-sensitivity C-reactive protein, and tumour necrosis factor-alpha. Family B (comprising lymphocyte-based ratios and composite indices) included the NLR, the platelet-to-lymphocyte ratio, the SII, the GLR, the lymphocyte-to-monocyte ratio, the systemic inflammation response index, and, as a pre-specified extension, the HALP (haemoglobin, albumin, lymphocyte, platelet) score. Since the HALP score is protective (higher values mean a better haematological and nutritional condition), its estimate was adjusted to correspond to the pro-risk direction and was expressed in terms of a one-SD deterioration so that it could be compared with the other markers. Markers of neuroaxonal injury or glial activation, such as neurofilament light chain and glial fibrillary acidic protein, were outside the scope of inflammation and therefore were not considered eligible. The outcome was PSCI as defined by a validated cognitive assessment tool (either the Montreal Cognitive Assessment [MoCA], the Mini-Mental State Examination or a neuropsychological battery) at a specific time point after stroke. It was necessary for the marker to have been measured before the cognitive outcome was established.

To ensure that exposure preceded outcome and to reduce selection and survivorship bias, inclusion was restricted to strictly prospective cohort studies. This criterion was pre-specified. Ambispective and registry-based analyses described by their authors as retrospective were excluded, as were case-control designs, cross-sectional analyses reporting only between-group differences in marker level, reviews, and animal and in vitro studies. Design was verified from each study’s own methods statement rather than from secondary labelling, because design descriptions in earlier reviews were unreliable. Studies had to report a covariate-adjusted association between the marker and PSCI, expressed as an odds ratio, hazard ratio or regression coefficient with a 95% confidence interval.

### 2.3 Information sources and search strategy

The search covered the databases of PubMed, Embase, Web of Science Core Collection, Scopus and the Cochrane Library from the time they were established up to June 2026; because of the language barrier the Chinese databases (CNKI and Wanfang) were not included. The search combined terms relating to stroke, to cognitive impairment and dementia, and to the individual markers and their families. The full search strings for each database are given in the Supplementary material. The reference lists of the studies that were included as well as those of earlier reviews were checked, and forward citation searches were carried out.

### 2.4 Study selection

The records were de-duplicated and then checked by title and abstract and at full text. The screening and coding were all carried out and verified by the three authors (BY, BCT, and NE) jointly, and agreement was reached on the inclusion decisions. A summary of the selection process is given in a PRISMA flow diagram (Figure 1).

**Figure 1:**
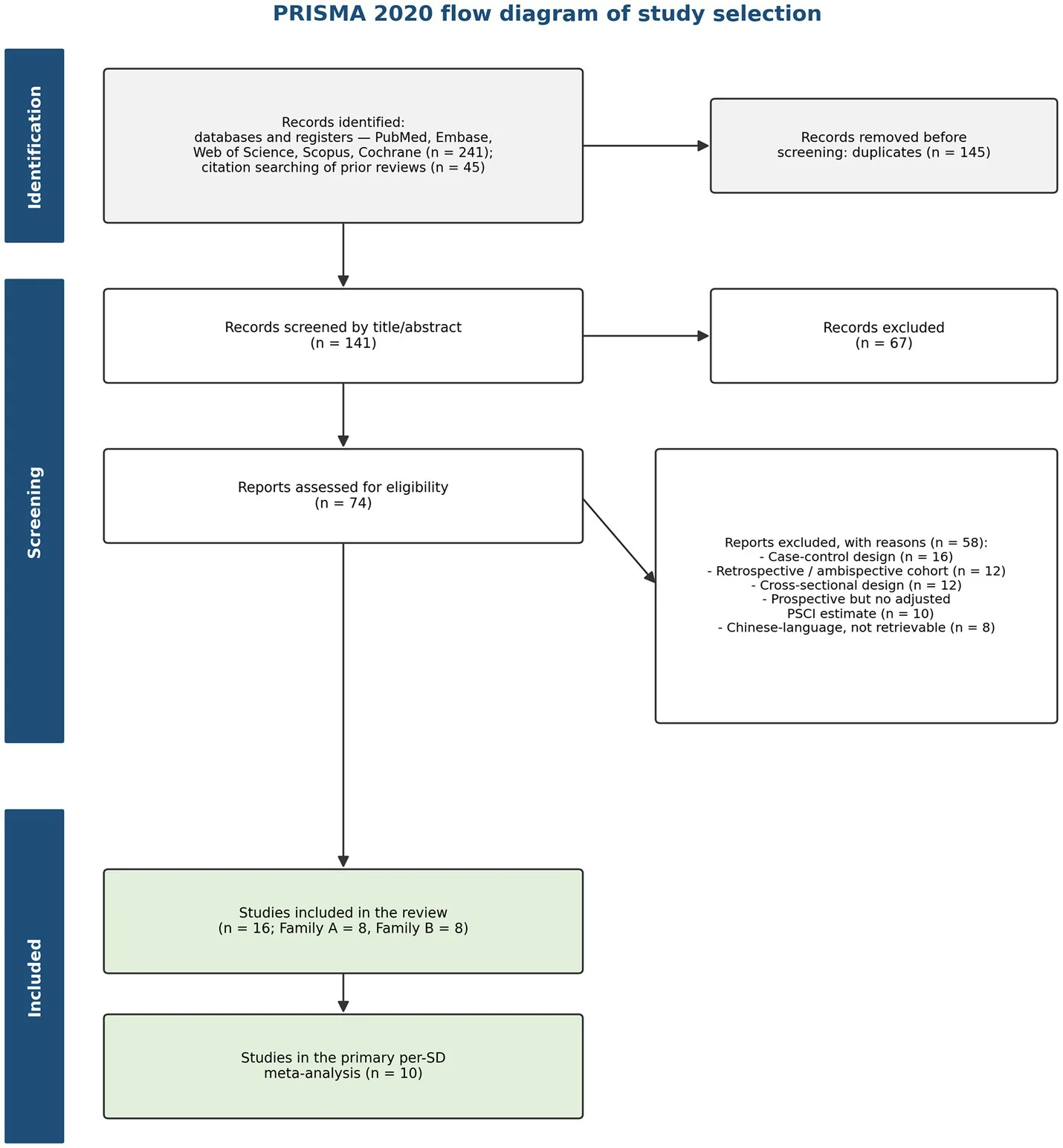
PRISMA 2020 flow diagram of study selection (16 studies included; 10 in the primary per-SD synthesis).

### 2.5 Data extraction

The data were obtained using a piloted form derived from CHARMS; all the data extraction and checking were done by the three authors, each entry being verified against the original source. The items that were extracted comprised the design and setting, the country, the sample size and the number of PSCI cases, the marker and its assay and the biological source, the timing of the measurement, the follow-up, the cognitive instrument and the definition of PSCI, the statistical model, the covariates that were adjusted for, the effect estimate together with its 95% confidence interval and scaling, and any diagnostic-accuracy metrics.

### 2.6 Effect-size harmonisation

The odds ratio for PSCI associated with one standard deviation (SD) increase in the marker was used as the main measure, enabling comparisons between estimates obtained on different scales. The effects were expressed on the natural-log scale, and the standard error was calculated from the reported confidence interval using the formula (ln(upper)−ln(lower))/(2 × 1.96). The estimates were set up in such a way that an odds ratio greater than one indicates higher odds of PSCI when the marker value is higher, and any protective associations were reversed.

The odds ratio given on a per-natural-unit basis was transformed into a per-SD scale by exponentiating the log odds ratio by the marker's standard deviation. When a study provided both a continuous (per-unit) estimate and a contrast between extreme categories, the continuous estimate was chosen for the main per-SD model since it does not depend on the assumption regarding the spacing of the categories, except in cases where the per-unit odds ratio was given to too few decimal places to allow for the conversion, in which situation the contrast between categories was used instead. This exception applied to one study (Cheng et al., 2024), since the per-unit odds ratio for the SII in that study was reported to three decimal places (1.002, 95% CI 1.001 to 1.002) and the marker's standard deviation was more than 400 units, so that rounding by itself would have produced a per-SD range extending from 1.87 to 2.83; in this case the tertile contrast was therefore used. As an extra sensitivity analysis, the three studies which had provided both types of estimate (Cheng et al., 2024; Huang et al., 2025; Lee et al., 2021) were re-analysed using the category-contrast method for all three. If a marker was presented as a median together with an interquartile range, the standard deviation was estimated using the method of Wan et al. (2014), and the subgroup summaries were combined to give an overall sample standard deviation. Since several of the markers are right-skewed, the per-SD conversions in this way are only approximate, and this point is looked at in the sensitivity analyses. In cases where a study gave only a contrast between extreme categories, the contrast was converted into a per-SD estimate by means of the category-spacing approach described by Chêne and Thompson (1996), using spacing factors of 2.18, 2.54, and 2.80 SD for tertiles, quartiles and quintiles respectively. The category contrasts were also kept on their original scale for the parallel highest-versus-lowest synthesis. To prevent double counting, each study supplied only one estimate for a given marker in the primary per-SD model. The regression coefficients obtained from the continuous cognitive scales could not be directly compared with the odds ratio estimates or with each other and were therefore summarised in a separate descriptive section.

### 2.7 Risk of bias

Risk of bias was assessed with QUIPS across its six domains: study participation, study attrition, prognostic factor measurement, outcome measurement, adjustment for other prognostic factors, and statistical analysis and reporting (Hayden et al., 2013). All risk-of-bias coding was carried out and checked by the three authors, with domain and overall ratings agreed by consensus. Domain and overall ratings are provided in Supplementary Table S2.

### 2.8 Statistical synthesis

The analyses were carried out using R and the metafor package (Viechtbauer, 2010); a detailed account of the computation can be found in the Supplementary material. In the case of markers having at least two independent estimates, the effects were combined using a random-effects model estimated by restricted maximum likelihood, and the confidence intervals and tests employed the Hartung-Knapp adjustment (Hartung and Knapp, 2001; IntHout et al., 2014). Since it was anticipated that the evidence within each marker would be limited, the main analysis involved a single model covering all the inflammatory estimates, with marker family (A compared with B) and marker serving as moderators, in order that the comparison between the families could be made within a single model rather than by making an informal comparison of the separate pooled estimates.

The original plan for ensuring that the results were not influenced by coinciding cohorts had provided for a three-level structure together with the use of cluster-robust variance estimation employing the CR2 small-sample correction, the cohort being the unit of clustering (Hedges et al., 2010; Pustejovsky and Tipton, 2018). As stated in Section 3.4, the main synthesis instead made use of a conventional random-effects model rather than adopting the planned multilevel and cluster-robust method. Heterogeneity in this model was measured using tau-squared, I-squared and Cochran’s Q; part of the planned multilevel approach had been the multilevel I-squared decomposition developed by Cheung (2014). A 95% prediction interval was computed (Higgins et al., 2009). Since the number of studies was small and there was a great deal of both clinical and statistical heterogeneity, formal small-study-effect tests were not carried out since the interpretation of such tests would be uncertain (Sterne et al., 2011). The pre-specified sensitivity analyses included leave-one-out estimation, the exclusion of those estimates obtained through the Chêne-Thompson conversion, the exclusion of the marker showing the most marked skew, and the exclusion of the pools based on two studies. In order to deal with the clinical heterogeneity relating to the definitions of PSCI and the follow-up period, we also excluded the cohorts in which cognition was assessed within 2 weeks of the stroke, since assessing it so early might capture acute confusional states rather than established PSCI and as a general rule the majority of consensus definitions require assessment to be carried out 3 months or later; we also looked at descriptive subgroups according to follow-up window (within 2 weeks, 3 months, 6 months or later) and according to cognitive instrument (MoCA-based versus other), and tested each moderator using meta-regression with Hartung-Knapp inference, acknowledging that these tests are underpowered given that there are only ten studies. The certainty was assessed for each marker using the GRADE approach as adapted for prognostic factors (Foroutan et al., 2020).

### 2.9 Diagnostic accuracy (secondary)

If at a defined threshold a sufficient number of studies had reported both sensitivity and specificity, then the diagnostic performance was to be summarised using a bivariate random-effects model and a hierarchical summary receiver operating characteristic curve (Reitsma et al., 2005); if this could not be done, the areas under the curve that had been reported were summarised in a narrative manner.

## 3 Results

### 3.1 Study selection

The database and register searches yielded 241 records, with an additional 45 being found through citation searching of earlier reviews. After the elimination of 145 duplicates, 141 records were evaluated on the basis of their title and abstract, 67 of which were excluded. Full texts were obtained for 74 reports, of which 58 were rejected for the following reasons: 16 had been designed as case-control studies, 12 were retrospective or ambispective cohorts, 12 were cross-sectional studies, 10 were prospective studies that provided no covariate-adjusted estimate for PSCI, and 8 were in Chinese and could not therefore be retrieved in full. Sixteen studies satisfied the eligibility criteria and were included (Family A, eight; Family B, eight; Figure 1). Ten of these studies reported an adjusted association that could be expressed as the odds ratio per standard deviation and so formed the basis of the primary synthesis; the rest contributed to the parallel highest-versus-lowest synthesis, to a descriptive continuous-cognition arm, or reported an adjusted association only in a subgroup, and are described narratively.

### 3.2 Study characteristics

The details of the studies included in the analysis are given in Table 1. The sizes of the analytic samples reported varied between 146 and 1,466 participants. The period of follow-up for the cognitive outcome extended from the first week following a stroke to several years. Generally, the cohorts were based at a single centre and were carried out in East Asia, although a smaller number were from Europe. The MoCA was most frequently used to define PSCI, the threshold employed varying (for example, a MoCA score below 22, 23, 25, or 26), and in one study cognition was assessed seven to 10ten days after the stroke. In the cytokine studies, IL-6 was most commonly examined, and in the ratio-index studies the SII, the NLR and the GLR were most often looked at. Although the sets of covariates differed from study to study, they usually included age, education, stroke severity and vascular risk factors.

**Table 1:** Characteristics of included studies.

Study	Country	Design	PSCI cases/analytic sample (n/N)	Family: marker	Timing of marker measurement	Follow-up	Cognitive outcome(instrument/definition)	Adjusted estimate reported and per-SDvalue used
Wang et al. (2022)	China	Prospective	–/1,003	A: IL-6	Acute	~1 y	MoCA-based	Q4 vs Q1 OR 1.95 (1.13–3.38); per-SD used: 1.30 (1.05–1.61), quartile contrast (CT)
Narasimhalu et al. (2015)	Singapore	Prospective	53/243	A: IL-6, IL-8, IL-12	Baseline	5 y	Cognitive decline	IL-6 ns; IL-8 OR 1.23; not in per-SD model
Sandvig et al. (2023) (Nor-COAST)	Norway	Prospective (multicentre)	–/455	A: IL-6	Acute	3–36 mo	MoCA	IL-6 β (age/sex adj); not in per-SD model
Giesers et al. (2025) (PROSCIS-B)	Germany	Prospective	–/570	A: IL-6	Baseline	1–3 y	TICS-m ≤ 31	IL-6 β −2.0 (−3.3 to −0.7); not in per-SD model
Carmichael et al. (2026) (Stroke-IMPaCT)	UK	Prospective (longitudinal)	NR	A: IL-6	≤96 h + serial	18–21 mo	Cognitive impairment	Per-doubling OR 8.16 (1.82–47.26); not in per-SD model
Guo et al. (2018)	China	Prospective	92/311 (ICAS)	A: CRP	Admission	6 mo	Six-item screener	Per-unit OR 1.038 (1.015–1.061); per-SD used: 1.59 (1.21–2.09), per-unit × SD 12.37
Chang et al. (2026)	China	Prospective	108/237	A: IL-6 (+Tregs)	Admission	~6 mo	MoCA/battery	Per-unit OR 1.269 (1.079–1.492); per-SD used: 2.96 (1.41–6.18), per-unit × SD 4.55
Cui et al. (2021)	China (CNSR-III)	Prospective	–/1466 (HHcy)	A: hs-CRP	Baseline	3 mo	MoCA	OR 1.42 (1.04–1.93), HHcy subgroup; not in per-SD model
Bao et al. (2023)	China	Prospective	130/254	B: SII	Admission	3 mo	MoCA	Per-SD OR 2.341 (1.44–3.81); per-SD used: 2.34 (1.44–3.81), reported per-SD
Cheng et al. (2024)	China	Prospective	193/332	B: SII (+SIRI)	Admission	3 mo	MoCA < 26	T3 vs T1 OR 10.37 (4.46–24.11); per-SD used: 2.92 (1.99–4.31), tertile contrast (CT)
Zhao et al. (2024)	China (Jilin)	Prospective (ChiCTR)	88/224	B: neutrophil%, NLR	Acute	6–12 mo	MoCA < 22	Neutrophil% OR 1.036 (1.004–1.069); not in per-SD model
Huang et al. (2025)	China	Prospective	71/146	B: GLR	Admission	7–10 d	MoCA < 23	T3 vs T1 OR 6.20 (2.10–18.33); per-SD used: 2.18 (1.27–3.75), per-unit × SD 7.48
Lee et al. (2021)	South Korea	Prospective (registry)	71/345	B: NLR	Admission	3 mo	Battery (z < −2 SD)	Q5 vs Q1 OR 3.26 (1.17–9.08); per-SD used: 1.13 (1.00–1.28), per-unit × SD 0.93
Zhang et al. (2024)	China (Jinzhou)	Prospective	101/254	B: NLR, PLR	Admission	3 mo	MoCA	NLR OR 1.437 (1.045–1.978); PLR OR 1.011 (1.001–1.021); per-SD used: 1.44 (1.05–1.98), per-unit × SD 1.00
Zuo et al. (2024)	China (multicentre)	Prospective (40 centres)	–/1022	B: HALP	Admission	12 mo	MoCA-B ≤ 22	Q1 vs Q4 OR 1.59 (1.07–2.37); per-SD used: 1.20 (1.03–1.40), quartile contrast (CT), direction-aligned
Xu et al. (2022)	China (Wenzhou)	Prospective (early-onset)	382/592	B: HALP	Admission	2 wk	MMSE	T1 vs T3 OR 1.965 (1.24–3.12); per-SD used: 1.36 (1.10–1.69), tertile contrast (CT), direction-aligned

Dashes denote counts not separately reported; the HALP score is protective, so low values indicate higher risk; NR, not reported; ICAS, intracranial arterial stenosis subgroup; HHcy, hyperhomocysteinaemia subgroup. CT, Chêne-Thompson category-spacing conversion (tertile /2.18, quartile /2.54, quintile /2.80). For Cheng et al. (2024), the per-unit odds ratio (1.002, 95% CI 1.001 to 1.002) was reported with insufficient precision for conversion, so the tertile contrast was used; for Huang et al. (2025) and Lee et al. (2021), the category route gives 2.31 (1.40 to 3.80) and 1.53 (1.06 to 2.20) respectively (Section 3.8).

### 3.3 Risk of bias

The QUIPS ratings for each study are provided in Supplementary Table S2; the most common issues were those concerning study participation, as most of the cohorts were based at a single centre, and problems relating to the measurement of prognostic factors, due to the skewed distribution of various markers and the reliance on a single baseline sample. Although the adjustment sets were usually adequate, they differed in the extent to which they included stroke severity and the burden of small-vessel disease.

### 3.4 Primary synthesis: odds of PSCI per standard deviation

In the ten separate cohorts, an increase of one standard deviation in the marker was linked to higher odds of PSCI, the pooled odds ratio being 1.60 (95% confidence interval 1.25 to 2.05; see Figure 2). There was a high degree of heterogeneity (tau-squared 0.081; I-squared 77%) and the 95% prediction interval ranged from 0.79 to 3.23, which meant that in certain situations the association might be nil. Since the ten cohorts originated from different source populations, the cluster-robust model was reduced to a standard random-effects analysis.

**Figure 2:**
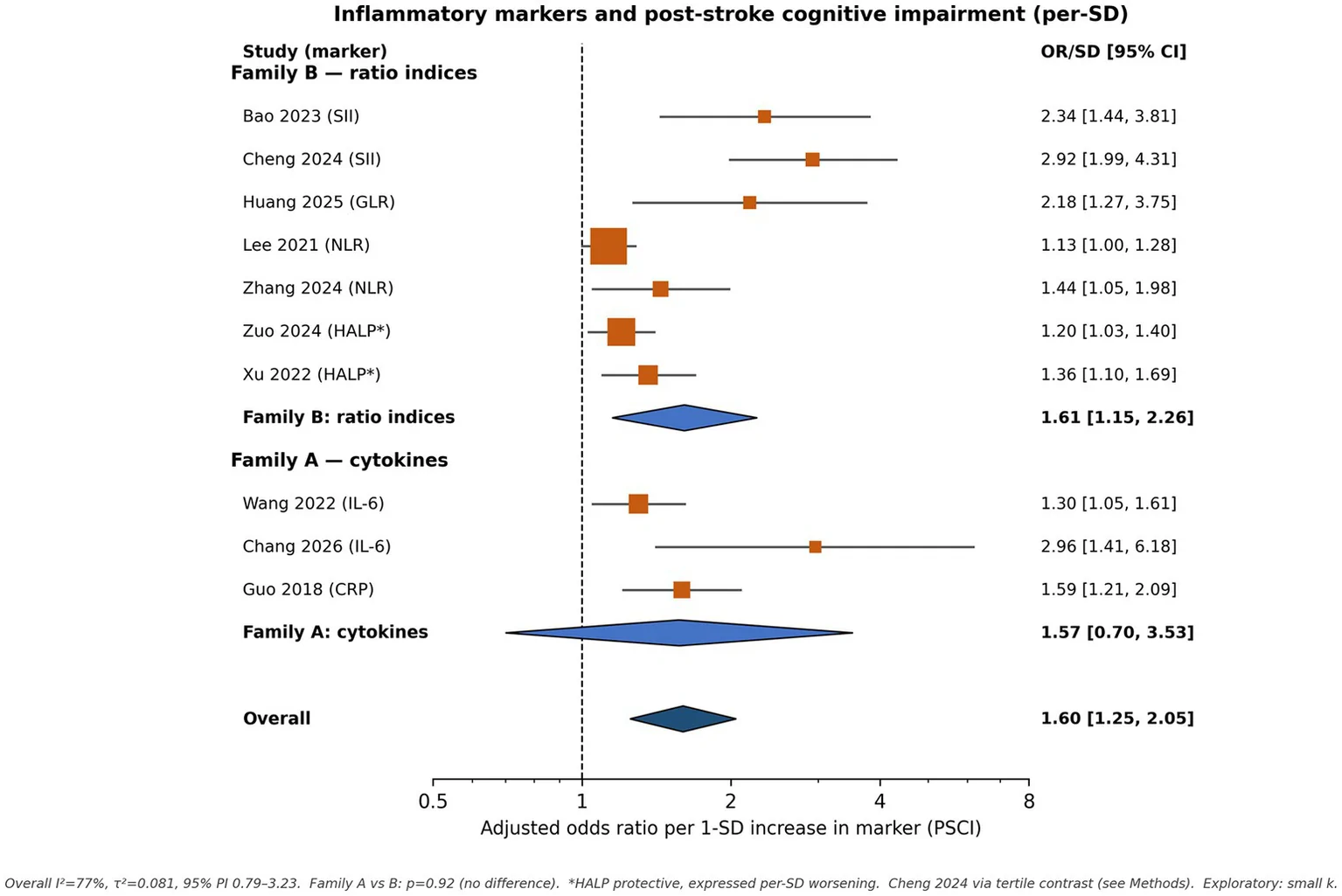
Forest plot of adjusted odds ratios for post-stroke cognitive impairment per one-standard-deviation increase in the marker, grouped by family, with family and overall pooled estimates. Squares are sized by inverse-variance weight.

When grouped by family, the pooled odds ratio was 1.61 (95% CI 1.15 to 2.26; I-squared 81%) for the ratio indices and 1.57 (95% CI 0.70 to 3.53) for the cytokines. For the individual markers, the two SII estimates were very close to each other (2.34 and 2.92 per SD), but the Knapp-Hartung interval from the two studies was extremely wide (pooled 2.68, 95% CI 0.68 to 10.62) and therefore provides no useful information; the point estimates for SII and GLR were higher than those for the other markers, even though each marker was based on only one or two cohorts (see Section 3.8). The NLR, which now included two cohorts, amounted to 1.22 (95% CI 0.30 to 4.99); the interval is wide because the two estimates—one close to the null and the other moderate—act in opposite directions. The two HALP estimates were in agreement and only modest (pooled 1.26, 95% CI 0.58 to 2.72) and when they were added together the ratio-index family estimate came nearer to that of the cytokines. The two IL-6 estimates showed considerable heterogeneity, ranging from 1.30 in the larger cohort to 2.96 in a smaller single-centre study, so the pooled IL-6 estimate was unstable. A single prospective study also gave the first in-scope adjusted estimate for the platelet-to-lymphocyte ratio (1.36 per SD), which was reported in the secondary synthesis. GLR and CRP were each based on a single study and so were not pooled.

### 3.5 Comparison between marker families

The pre-specified comparison showed that there was no statistically significant difference in the estimated per-SD associations among the marker families. The ratio of the odds ratios for the ratio indices as compared with those for the cytokines was 0.97 (95% CI 0.54 to 1.77; *t* = −0.10, df = 8, *p* = 0.92). Because of the wide confidence interval, there remains considerable uncertainty regarding the relative strength of these associations and no conclusion can be drawn as to their similarity.

### 3.6 Highest-versus-lowest synthesis

The results of the parallel synthesis, which was based on contrasts between extreme categories, were in agreement with those of the per-SD analysis. When the highest category was compared with the lowest one, the adjusted odds ratio for PSCI was 10.37 in the case of SII (tertiles), 6.20 for GLR (tertiles), 3.26 for NLR (quintiles) and 1.95 for IL-6 (quartiles). Since each of the contrasts involved a different marker, the estimates were not combined and are given here for descriptive purposes.

### 3.7 Continuous-cognition and subgroup findings

Studies that based their model of cognition on a continuous scale found an inverse relationship between inflammation and cognitive performance, but these studies could not be combined. In two European cohorts, higher levels of IL-6 were linked to lower subsequent cognitive scores on the MoCA and on a cognitive assessment carried out by telephone (Sandvig et al., 2023; Giesers et al., 2025). In one of the cohorts an adjusted odds ratio of 8.16 was observed for each doubling of IL-6 regarding cognitive impairment (Carmichael et al., 2026). Two additional cytokine findings were either restricted or showed no effect: the adjusted association with high-sensitivity CRP was seen only in the subgroup with hyperhomocysteinaemia (odds ratio 1.42), and in one long-term cohort there was no independent association found for IL-6 although associations were reported for declines in interleukin-8 and interleukin-12 (Narasimhalu et al., 2015). The results are in line with a real but heterogeneous cytokine signal affecting cognition, even though this signal is less prominent than the amount of research into stroke-related cytokines would indicate.

### 3.8 Sensitivity analyses

The pooled per-SD estimate was robust to the analytical choices examined. In leave-one-out analysis, the overall odds ratio remained between 1.44 and 1.68 regardless of which study was removed. Excluding the four estimates obtained through the Chêne-Thompson category conversion gave 1.67 (95% CI 1.16 to 2.40), and excluding the marker with the most pronounced skew (CRP) gave 1.62 (95% CI 1.23 to 2.14). Within the ratio-index family, heterogeneity was driven by the lower estimates for the NLR and the HALP score: the SII and GLR estimates, from three cohorts in total, clustered at approximately 2.5 per SD with no detectable heterogeneity (pooled 2.55, 95% CI 1.43 to 4.56; I-squared 0%), whereas removing the near-null NLR estimate alone gave 1.73 (95% CI 1.18 to 2.54) with heterogeneity persisting. Because three studies reported both a per-unit estimate and an extreme-category contrast, the analysis was repeated using the category-contrast route for all three (Cheng et al., 2024; Huang et al., 2025; Lee et al., 2021). The two routes agreed closely for the GLR (2.18 versus 2.31 per SD) but diverged for the NLR (1.13 versus 1.53 per SD), which indicates a non-linear association concentrated in the highest NLR quintile. Under this alternative route, the overall estimate was 1.67 (95% CI 1.33 to 2.08; I-squared 71%), the ratio-index family estimate was 1.70 (95% CI 1.24 to 2.33), the two NLR estimates became concordant (1.48 per SD; I-squared 0%), and the family comparison remained null (*p* = 0.85). Clinical heterogeneity in the definition and timing of PSCI was examined directly. Two cohorts assessed cognition within 2 weeks of stroke (at 7 to 10 days and at 2 weeks); excluding them left the pooled estimate unchanged (1.61, 95% CI 1.19 to 2.18; I-squared 80%), and the moderator test for early versus later assessment was null (ratio of odds ratios 1.02, 95% CI 0.51 to 2.01; *p* = 0.96). Estimates were directionally consistent across follow-up windows, at 1.77 (95% CI 0.87 to 3.61) for the four cohorts assessed at 3 months and 1.39 (95% CI 1.02 to 1.91) for the four assessed at 6 months or later, with overlapping intervals. Estimates from the seven cohorts using the MoCA (1.80, 95% CI 1.25 to 2.59) were numerically higher than those from the three using other instruments (1.31, 95% CI 0.85 to 2.01), but the difference was not significant (*p* = 0.23). Heterogeneity remained high within every subgroup, so differences in PSCI definition and follow-up account for only part of the between-study variation. Consistent with the pre-specified plan and the small number of studies, funnel-plot asymmetry and regression tests were not performed; the funnel plot is provided for description only (Figure 3).

**Figure 3:**
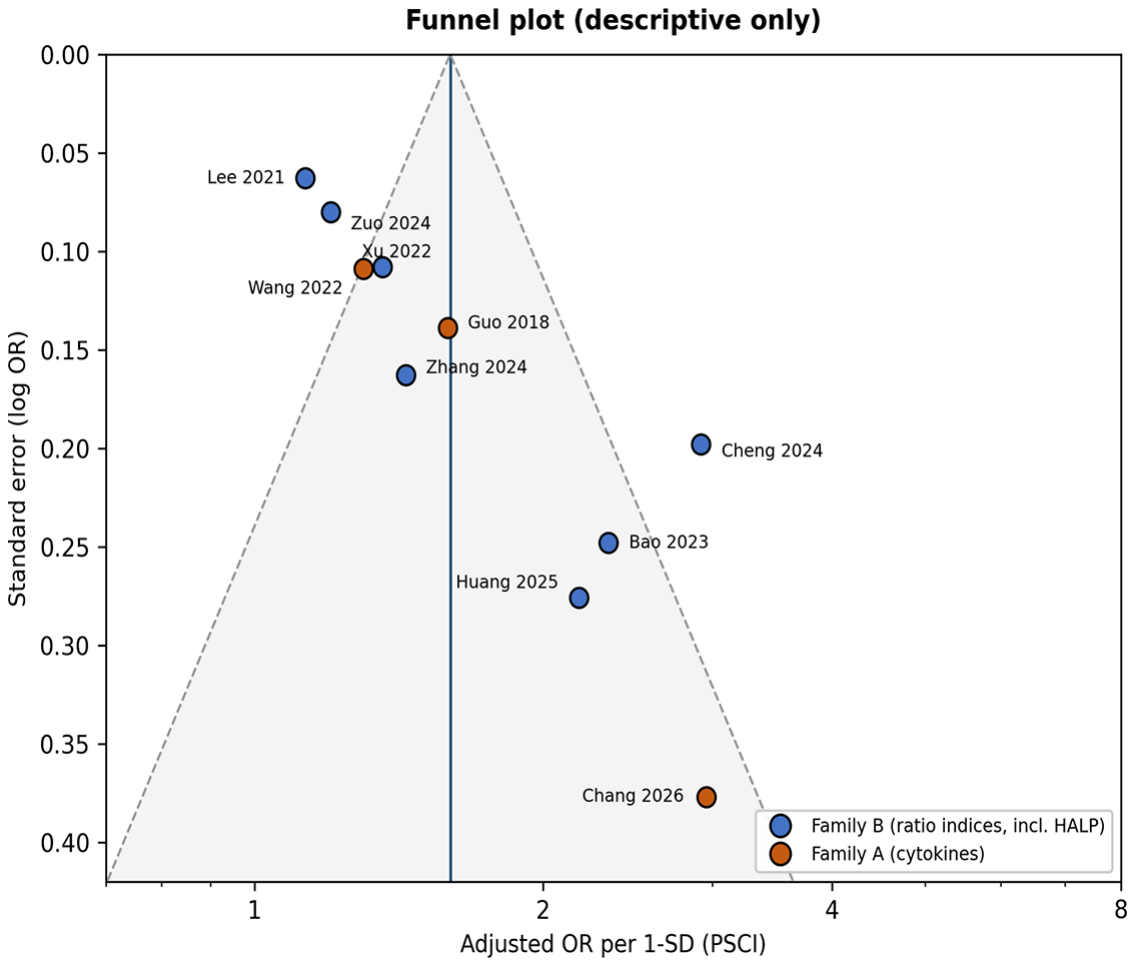
Funnel plot of the per-standard-deviation estimates, presented for description only. Given the ten studies and substantial heterogeneity, formal tests of funnel asymmetry were not performed because their interpretation would be uncertain.

## 4 Discussion

In the systematic review and meta-analysis involving only prospective cohorts, higher levels of circulating inflammatory markers were linked to increased adjusted odds of PSCI according to the overall pooled estimate (with an odds ratio of 1.60 for each standard deviation). However, the association showed heterogeneity and the prediction interval included the null value, meaning that the finding is only suggestive and not firmly established, and the effect varies from one setting to another. When the two pre-specified groups were compared, no statistically significant difference in the estimated associations was found. Yet the wide confidence interval does not prove equivalence nor does it rule out a meaningful difference. The point estimates for SII and GLR were higher than those for the other markers, but only one or two cohorts provided data for each of these markers, so this order is only provisional; the lower estimates for NLR and the HALP score account for much of the heterogeneity observed within the ratio-index family. The contribution of this study is comparative rather than confirmatory: while the common adjusted per-SD scale allows for an exploratory comparison of the associations, the evidence is too limited and too heterogeneous to enable a reliable ranking of the candidate markers. Differences between the populations and the adjustment models also hinder the comparisons, and the magnitude of the associations does not directly indicate comparative predictive performance.

The different markers stand for various aspects of systemic inflammation; the SII involves the counts of neutrophils, platelets and lymphocytes, while the NLR shows the relationship between neutrophils and lymphocytes. The GLR uses measurements of globulin and lymphocyte levels. Although these biological differences suggest areas for further investigation, the current estimates are based on one or two cohorts for each marker and therefore do not allow for a ranking. The fact that the comparison between families is not significant means that it is uncertain which family has a stronger association with PSCI and it does not prove that their predictive performance is similar.

It is worth highlighting the findings regarding cytokines. Although IL-6 and CRP are frequently referred to as the best-studied inflammatory markers in the context of stroke, most of the evidence available relates to vascular recurrence and major adverse cardiovascular events rather than to cognitive function (McCabe et al., 2023). If one focuses only on adjusted, prospective studies that are specific to cognition, the evidence for IL-6 is weak and varied: a large cohort study found a small association per standard deviation, a smaller single-centre study found a strong association, one long-term cohort found no independent effect of IL-6 although it did point to the role of other cytokines, and two European cohorts analysed IL-6 in relation to continuous cognition scores that could not be combined. Therefore, the apparent maturity of the cytokine literature in stroke does not result in solid cognition-specific evidence.

The results extend and refine previous syntheses. A recent meta-analysis of lymphocyte-associated indices found higher marker levels in patients with PSCI by combining the unadjusted mean differences (Mao et al., 2025), and broader reviews of biomarkers had likewise used an approach based on differences in levels (Kim et al., 2022; Jiang et al., 2025). In this study, by limiting the inclusion to the adjusted estimates from prospective cohorts and by converting the measures to per-SD odds ratios, a prognostic question has been asked rather than a descriptive one and a more conservative conclusion has been reached. The importance of distinguishing between biomarker questions is also illustrated by a recent meta-analysis of circulating BDNF in paediatric neurodevelopmental disorders, which separately examined case–control differences and associations with cognitive impairment severity within affected populations (Çapa Tayyare et al., 2026). The difference from the earlier, more optimistic summaries is to be expected since unadjusted differences in levels can be influenced by confounding factors such as age, education and stroke severity, all of which are related to both inflammation and cognition.

The meta-analysis has a number of advantages. By adopting a strictly prospective design, it ensures that exposure came before the outcome and thus minimises the problems of reverse causation and survivorship bias. Only the adjusted estimates were combined; the cross-family comparison had been previously specified and formally tested; a cluster-robust method was specified in relation to cohort overlap, even though the primary synthesis reported used conventional random-effects analysis; and all the scale conversions were recorded and looked at in the sensitivity analyses. The overall estimate remained stable when each study was left out in turn and was not influenced by any single study or by the category and skew-related conversions.

The conclusions are subject to several limitations. The number of studies available for each marker was small, the majority of the syntheses being based on one or two cohorts only, and as a result inferences made within each marker are restricted and the confidence intervals derived from pools of two studies are unreliable and extremely wide. The process of converting the reported estimates into per-SD terms depends on the form in which each study presented its results: in cases where a per-unit odds ratio is given with only a few decimal places and the marker has a large standard deviation, as was the case with the SII in one study, simple rounding can cover a broad per-SD range, and when the association is non-linear, as indicated by the difference between the per-unit and the extreme-quintile approaches for the NLR, no single per-SD value is entirely appropriate. These problems were dealt with by applying the estimate-selection rule and by re-analysing the relevant studies using the alternative method, a procedure which did not alter the conclusions. Most of the cohorts were based at a single centre and were carried out in East Asia, which limits the generalisability of the findings. Some of the markers are right-skewed, so the estimation of standard deviations from medians and interquartile ranges and the conversion of category contrasts to a per-SD scale have to be approximate; these approximate values were checked and had no significant effect on the pooled estimate. The studies included used a variety of cognitive assessment tools (the MoCA at thresholds ranging from 22 to 26, the MMSE, a neuropsychological battery and a brief screener) and had follow-up periods ranging from 7 to 10 days up to more than a year. Two of the cohorts assessed cognition within 2 weeks of the stroke, a time when the impairment might be due to acute confusional effects rather than to established PSCI. Omitting these two cohorts had no effect on the pooled estimate, and the results for the follow-up and instrument subgroups were in the same direction. Nevertheless, a high level of heterogeneity remained within each subgroup, so clinical heterogeneity in how the outcome was defined accounts for only part of the variation between the studies, and the remaining heterogeneity represents a real limitation of the current body of evidence. The search did not include Chinese-language databases, so some relevant prospective cohorts may have been overlooked. Formal tests for small-study effects were not carried out because the small size of the evidence base and the considerable heterogeneity would have made any interpretation of such tests unreliable, and the review was not registered. Several of the cohorts included both ischaemic stroke and transient ischaemic attack (for example, the ICONS, CNSR-III and Nor-COAST samples), and since too few studies provided separate estimates for transient ischaemic attack, a stratified analysis could not be performed; hence it was not possible to examine any difference in the association between the marker and PSCI for ischaemic stroke and transient ischaemic attack.

In clinical practice, this review does not demonstrate the clinical usefulness of any inflammatory marker for use in the stratification of PSCI risk. Even though the SII and GLR had relatively high point estimates, the limited amount of evidence is not sufficient to rank them above the other markers. For research purposes, the priority should be to conduct multi-centre prospective cohort studies outside East Asia in which several markers are measured in the same participants, use standardised definitions of PSCI and standard follow-up periods, and report pre-specified adjusted models in order that the markers can be directly compared. A synthesis using individual participant data would deal with several of the limitations mentioned here.

## 5 Conclusion

Several prospective cohorts have found that higher levels of inflammation-related biomarkers measured after a stroke are linked to an increased likelihood of later developing cognitive impairment, but there is considerable clinical and statistical variability among the studies: the pooled odds ratio per standard deviation was 1.60 (95% CI 1.25 to 2.05), the I-squared value was 77%, the prediction interval included the null result, and the ten cohorts involved were mostly based in single centres in East Asia and used different cognitive assessment tools, thresholds, and follow-up periods. Within these constraints, three conclusions can be made with precision. The first is that the comparison did not identify a statistically significant difference between the adjusted associations for lymphocyte-based ratio indices and cytokines (the ratio of odds ratios being 0.97, 95% CI 0.54 to 1.77), although the confidence interval is so wide that equivalence cannot be established. The second point is that the estimates for individual markers, such as SII and GLR, are based on one or two cohorts only and therefore do not allow for a reliable ranking or prioritisation. Third, this review does not prove that circulating inflammatory markers have clinical usefulness for the risk stratification of PSCI; instead, standardised, multi-centre prospective studies are required to assess their predictive performance and additional value.

## Data Availability

The original contributions presented in the study are included in the article/Supplementary material, further inquiries can be directed to the corresponding author.
