# Supplementary material for: Circulating inflammatory biomarkers as adjusted prognostic predictors of post-stroke cognitive impairment: a comparative meta-analysis of cytokines and lymphocyte-based ratio indices in prospective cohorts
Source: Front Aging Neurosci. 2026 Oct 21;18:1937870. doi: 10.3389/fnagi.2026.1937870 (PMC13760503; doi:10.3389/fnagi.2026.1937870)
Supplement: Supplementary file 1 [file Table_1.docx]

# Supplementary Table S1. Included studies

Reference list of the studies included in the systematic review and meta-analysis.

## Family A — cytokines and acute-phase proteins

Carmichael, N. S., Deijnen, H. R., Wong, S. Y., Williams, T. O., Kontopantelis, E., Cowie, L., Jones, E., Drag, L., Buckwalter, M. S., Grainger, J. R., Allan, S. M., & Smith, C. J. (2026). Longitudinal plasma interleukin-6 and post-stroke cognitive outcomes: The Stroke-IMPaCT study. *Alzheimer’s & Dementia, 22*(3), e71261. https://doi.org/10.1002/alz.71261

Chang, Y., Zhang, X., Yu, C., Zhu, L., Sun, S., & Sun, Z. (2026). Peripheral blood regulatory T cells and IL-6 are associated with cognitive impairment after acute ischemic stroke. *Neuropsychiatric Disease and Treatment, 22*, 590109. https://doi.org/10.2147/NDT.S590109

Cui, L., Lu, P., Li, S., Pan, Y., Wang, M., Li, Z., Liao, X., & Wang, Y. (2021). Relationship among homocysteine, inflammation and cognitive impairment in patients with acute ischemic stroke and transient ischemic attack. *Neuropsychiatric Disease and Treatment, 17*, 3607–3616. https://doi.org/10.2147/NDT.S333753

Giesers, N. K., Schaeff, V., Gertz, K., Endres, M., & Liman, T. G. (2025). Associations of soluble inflammatory and endothelial activation biomarkers with cognitive function over three years after ischemic stroke: PROSCIS-B. *Translational Stroke Research, 16*(6), 2249–2257. https://doi.org/10.1007/s12975-025-01388-4

Guo, J., Su, W., Fang, J., Chen, N., Zhou, M., Zhang, Y., & He, L. (2018). Elevated CRP at admission predicts post-stroke cognitive impairment in Han Chinese patients with intracranial arterial stenosis. *Neurological Research, 40*(4), 292–296. https://doi.org/10.1080/01616412.2018.1438224

Narasimhalu, K., Lee, J., Leong, Y. L., Ma, L., De Silva, D. A., Wong, M. C., Chang, H. M., & Chen, C. (2015). Inflammatory markers and their association with post-stroke cognitive decline. *International Journal of Stroke, 10*(4), 513–518. https://doi.org/10.1111/ijs.12001

Sandvig, H. V., Aam, S., Alme, K. N., Askim, T., Beyer, M. K., Ellekjær, H., Ihle-Hansen, H., Lydersen, S., Mollnes, T. E., Munthe-Kaas, R., Næss, H., Saltvedt, I., Seljeseth, Y. M., Thingstad, P., Wethal, T., & Knapskog, A.-B. (2023). Plasma inflammatory biomarkers are associated with poststroke cognitive impairment: The Nor-COAST study. *Stroke, 54*(5), 1303–1311. https://doi.org/10.1161/STROKEAHA.122.041965

Wang, Y., Li, J., Pan, Y., Wang, M., Lin, J., Meng, X., Liao, X., & Wang, Y. (2022). Interleukin-6 as predictor of one-year cognitive function after ischemic stroke or TIA. *Neuropsychiatric Disease and Treatment, 18*, 391–399. https://doi.org/10.2147/NDT.S348409

## Family B — lymphocyte-based ratio and composite indices

Bao, Y., Wang, L., Du, C., Ji, Y., Dai, Y., & Jiang, W. (2023). Association between systemic immune-inflammation index and cognitive impairment after acute ischemic stroke. *Brain Sciences, 13*(3), 464. https://doi.org/10.3390/brainsci13030464

Cheng, Y., Zhu, H., Liu, C., Li, L., Lin, F., Guo, Y., Gu, C., Sun, D., Gao, Y., He, G., Sun, S., & Xue, S. (2024). Systemic immune-inflammation index upon admission correlates to post-stroke cognitive impairment in patients with acute ischemic stroke. *Aging, 16*(10), 8810–8821. https://doi.org/10.18632/aging.205839

Huang, W., Liao, L., Liu, Q., Ma, R., Hu, W., Dai, Y., Wang, L., & Sha, D. (2025). Predictive value of circulating inflammatory biomarkers for early-onset post-stroke cognitive impairment: A prospective cohort study. *Frontiers in Neurology, 16*, 1565613. https://doi.org/10.3389/fneur.2025.1565613

Lee, M., Lim, J.-S., Kim, C.-H., Lee, S.-H., Kim, Y., Lee, J.-H., Jang, M. U., Oh, M. S., Lee, B.-C., & Yu, K.-H. (2021). High neutrophil-lymphocyte ratio predicts post-stroke cognitive impairment in acute ischemic stroke patients. *Frontiers in Neurology, 12*, 693318. https://doi.org/10.3389/fneur.2021.693318

Xu, M., Chen, L., Hu, Y., Wu, J., Wu, Z., Yang, S., Kang, W., He, J., & Ren, W. (2022). The HALP (haemoglobin, albumin, lymphocyte, and platelet) score is associated with early-onset post-stroke cognitive impairment. *Neurological Sciences, 44*(1), 237–245. https://doi.org/10.1007/s10072-022-06414-z

Zhang, S. (2024). The correlation between neutrophil-to-lymphocyte ratio and cognitive impairment in patients with acute cerebral infarction [in Chinese]. *Journal of Shanxi Datong University (Natural Science Edition), 40*(2), 91–95. https://doi.org/10.3969/j.issn.1674-0874.2024.02.017

Zhao, P., Zhang, G., Wang, Y., Wei, C., Wang, Z., Zhai, W., Shen, Y., Shi, L., & Sun, L. (2024). Peripheral immunity is associated with cognitive impairment after acute minor ischemic stroke and transient ischemic attack. *Scientific Reports, 14*, Article 16201. https://doi.org/10.1038/s41598-024-67172-w

Zuo, L., Dong, Y., Liao, X., Hu, Y., Pan, Y., Yan, H., Wang, X., Zhao, X., Wang, Y., Seet, R. C. S., Wang, Y., & Li, Z. (2024). Low HALP (haemoglobin, albumin, lymphocyte, and platelet) score increases the risk of post-stroke cognitive impairment: A multicentre cohort study. *Clinical Interventions in Aging, 19*, 81–92. https://doi.org/10.2147/CIA.S432885
