# Supplementary material for: Circulating inflammatory biomarkers as adjusted prognostic predictors of post-stroke cognitive impairment: a comparative meta-analysis of cytokines and lymphocyte-based ratio indices in prospective cohorts
Source: Front Aging Neurosci. 2026 Oct 21;18:1937870. doi: 10.3389/fnagi.2026.1937870 (PMC13760503; doi:10.3389/fnagi.2026.1937870)
Supplement: Supplementary file 2 [file Table_2.docx]

# Supplementary Table S2. Risk of bias (QUIPS) for included studies

Assessment with the Quality In Prognosis Studies (QUIPS) tool across six domains: (1) study participation, (2) study attrition, (3) prognostic factor measurement, (4) outcome measurement, (5) adjustment for other prognostic factors (confounding), and (6) statistical analysis and reporting. Ratings are Low, Moderate or High risk.

| Study | 1. Participation | 2. Attrition | 3. PF measurement | 4. Outcome | 5. Confounding | 6. Analysis/reporting | Overall |
| --- | --- | --- | --- | --- | --- | --- | --- |
| Wang 2022 (IL-6) | Moderate (single registry) | Low | Low | Low (MoCA) | Low (broad adjustment) | Moderate | **Moderate** |
| Narasimhalu 2015 (cytokines) | Low (cohort) | Moderate (5-y losses?) | Low | Low (battery) | Moderate | Moderate (null for IL-6) | **Moderate** |
| Sandvig 2023 (Nor-COAST, IL-6) | Low (multicentre) | Low | Low | Low (MoCA, blinded) | High (age/sex only) | Moderate | **Moderate–High** |
| Giesers 2025 (PROSCIS-B, IL-6) | Low | Moderate (attrition over 3 y) | Low (log-transformed) | Low (TICS-m) | Low (rich adjustment) | Low | **Low–Moderate** |
| Carmichael 2026 (Stroke-IMPaCT, IL-6) | Moderate (single programme) | Moderate (serial sampling) | Low | Moderate (mixed outcomes) | Moderate | Moderate | **Moderate** |
| Guo 2018 (CRP) | High (ICAS subgroup only) | Moderate | Moderate (CRP skew, acute-phase) | Moderate (six-item screener) | Moderate (subgroup) | Moderate | **Moderate–High** |
| Chang 2026 (IL-6) | Moderate (single centre) | Moderate | Moderate (IL-6 skew) | Low (MoCA/battery) | Moderate | Moderate (PSD subset) | **Moderate** |
| Cui 2021 (hs-CRP) | Moderate (CNSR-III subcohort) | Low | Moderate (skew) | Low (MoCA) | High (HHcy subgroup, selection) | Moderate | **Moderate–High** |
| Bao 2023 (SII) | Moderate (single centre) | Low | Moderate (SII skew) | Low (MoCA) | Moderate | Low | **Moderate** |
| Cheng 2024 (SII) | Moderate (single centre) | Moderate | Moderate (SII skew) | Low (MoCA<26) | Low (broad adjustment) | Low | **Moderate** |
| Zhao 2024 (neutrophil%/NLR) | Low (registered, ChiCTR) | Low | Moderate (fractions) | Low (MoCA) | Moderate (NLR subgroup) | Moderate | **Moderate** |
| Huang 2025 (GLR) | Moderate (single centre) | Low | Moderate (GLR skew) | Moderate (very early, 7–10 d) | Low (broad adjustment) | Low | **Moderate** |
| Lee 2021 (NLR) | Moderate (registry) | Moderate | Low | Moderate (battery cut-off) | Moderate | Moderate | **Moderate** |
| Zhang 2024 (NLR) | Moderate (single centre) | Moderate (attrition not detailed) | Low | Moderate (MMSE) | Moderate (adjustment set not stated in source) | Moderate | **Moderate** |
| Zuo 2024 (HALP) | Low (multicentre, 40 sites) | Moderate (12-mo attrition) | Low | Low (MoCA-Beijing) | Moderate (protective marker; direction-aligned) | Low | **Low–Moderate** |
| Xu 2022 (HALP) | Moderate (single centre; prospective design to confirm) | Moderate | Low | Moderate (MMSE, 2-wk early) | Moderate | Moderate | **Moderate** |
